# Incidence of lower extremity amputation in the diabetic compared to the non-diabetic population: a systematic review protocol

**DOI:** 10.1186/s13643-015-0064-9

**Published:** 2015-05-23

**Authors:** Tatjana Kvitkina, Maria Narres, Heiner Claessen, Sigrid Droste, Stephan Morbach, Oliver Kuss, Andrea Icks

**Affiliations:** Department of Public Health, Centre of Health and Society, Heinrich-Heine-University, Düsseldorf, Germany; Institute of Biometrics and Epidemiology, German Diabetes Centre, Leibniz Centre for Diabetes Research, Heinrich-Heine-University Düsseldorf, Auf’m Hennekamp 65, 40225 Düsseldorf, Germany; Department of Diabetes and Angiology, Marienkrankenhaus, Soest, Germany

**Keywords:** Diabetes, Population-based study, Incidences, Lower extremity amputations (LEA), Systematic review, Protocol

## Abstract

**Background:**

Diabetic individuals have a largely increased risk of lower extremity amputation (LEA) compared with non-diabetic patients. Prior systematic reviews of incidence of LEA have some limitations with respect to lack of consensus in the definition of LEA, level of LEA (all, major, minor), and definition of source population (general population or population with diabetes at risk). The purpose of our review is to evaluate the incidence of LEA in the diabetic population and its differences with regard to sex, ethnicity, age, and regions; to compare the incidence rate (IR) in the diabetic and non-diabetic population; and to investigate time trends.

**Methods/design:**

We will perform a systematic literature search in MEDLINE, Embase, Web of Knowledge, and publisher databases such as Journals@OVID and ScienceDirect. We will develop comprehensive systematic search strategies according to established guidelines for meta-analyses of observational studies in epidemiology (the MOOSE group). Two authors will independently screen abstracts and full text of all references on the basis of inclusion criteria with respect to types of study, types of population, and the main outcome. We will exclude studies if they report solely incidences of LEA among persons with diabetes mellitus when referring to the total population (diabetic and non-diabetic) and not exclusively to the diabetic population. Data extraction and assessment of risk of bias will be undertaken by two review authors working independently. We will assess incidence rate (IR) or cumulative incidence (CumI), relative risk of amputations comparing the diabetic to non-diabetic populations, cause of LEA, and type of diabetes. If we find subsets of studies to be homogeneous enough, we will perform meta-analyses for incidence rates by Poisson generalized linear mixed models (GLMM).

**Systematic review registration:**

PROSPERO CRD42015017809

**Electronic supplementary material:**

The online version of this article (doi:10.1186/s13643-015-0064-9) contains supplementary material, which is available to authorized users.

## Background

The prevalence of diabetes mellitus has increased substantially and has reached 8.3 % in 2014 which corresponds to 387 million patients globally [1]. This overall increment leads to the growth in the number of individuals with diabetic complications including peripheral arterial disease, peripheral neuropathy, and lower extremity amputation (LEA).

In the Western countries, LEA has frequently been cited as a primary objective by health systems and organization [2], and diabetic individuals still have a largely increased LEA risk compared with non-diabetic patients [3, 4]. According to Vamos et al. and Trautner et al., people with diabetes have up to a 40-fold increased risk of LEA when compared with the general population, and also approximately half of all people undergoing non-traumatic amputations are diagnosed with diabetes [5, 6]. Avoidance of amputation should not only be targeted because of the associated economic consequences (high costs due to repeated hospitalizations, rehabilitation, home care, and social-service support) but also due to quality-of-life issues [7].

A number of reviews have summarized the published medical literature on the incidence of LEA in diabetic and non-diabetic populations (Moxey et al. [8], Ephraim et al. [9], Larsson et al. [10], Pernot et al. [11]). However, all of these have some limitations, especially (1) lack of consensus in the definition of “lower extremity amputation” with respect to the cause of LEA as well as in the reporting of incidence of LEA (one or more events per person), (2) level of LEA (all, major, minor), (3) with respect to selection of the study population, and (4) definition of source population (general population or population with diabetes at risk).

Given the lack of systematic knowledge, we will conduct a first systematic review concerning incidence of LEA in diabetic patients referred to a population at risk.

### Objectives

The main objectives of this review are (1) to evaluate the incidence of LEA in the diabetic population and differences between incidences of LEA with respect to sex, ethnicity, age, and regions; (2) to compare incidence rates of LEA in the diabetic and non-diabetic population; and (3) to investigate time trends.

This systematic review is part of an ongoing national initiative, aimed to evaluate time trends of amputation, end-stage renal disease, blindness, stroke, myocardial infarction, and adverse perinatal outcome [12].

## Methods and design

The proposed review protocol conforms to the PRISMA-P guidelines [13].

### Eligibility criteria

#### Types of studies

All population-based longitudinal studies using both prospective and retrospective designs should be included for this review.

#### Types of populations

The source populations should firstly be defined by official statistics, which means e.g., all inhabitants of a defined region or all insured persons of a statutory health insurance. Secondly, all the included individuals with diabetes (incident or prevalent) should be known or estimated in a valid manner. Hence, source population which we will study could be (1) general population, divided into those with and those without diabetes, (2) all individuals with prevalent diabetes within a defined population, or (3) all individuals with incident diabetes within a defined population.

Individuals with diabetes can have type 1 diabetes, type 2 diabetes, or diabetes without definition of diabetes type. We will also consider old diabetes classifications, namely insulin-dependent (IDDM) and non-insulin-dependent diabetes mellitus (NIDDM). Individuals without diabetes will also be considered with the aim of comparing incidences between diabetic und non-diabetic populations.

#### Outcomes

The main outcome should be the incidence of LEA among patients with diabetes mellitus (DM). The analysis of incidence LEA will be done according to the following parameters:

- ■ Epidemiologic incidence measures:
  - □ Incidence rate (IR)
  - □ Cumulative incidence (CumI)
- ■ Reporting incidence of LEA:
  - □ Person level (only one amputation per person).
  - □ Case level, e.g., from hospital data (on the same admission only one amputation per person). This could be several hospitalizations per person in the same calendar year.
  - □ Procedure level (all amputations).
- ■ Level of LEA:
  - □ All (independent of level)
  - □ Major and minor amputations

Different definitions of amputations (major, minor, or all) can be used in the studies. We will describe the respective definitions of LEA level of these studies.

Moreover, we will consider secular time trends as well as differences in the LEA risk between demographic variables (sex, ethnicity, age) and regions. Additionally, we will investigate relative risks (RR), comparing the incidence of LEA among populations with and without diabetes.

### Information sources

We will perform a systematic literature search in MEDLINE, Embase, Web of Knowledge, and publisher databases such as Journals@OVID and ScienceDirect. Moreover, we will use other resources to search potentially eligible studies, such as reference lists of review articles and relevant studies. We will contact authors of such potential studies for full text if the full text is otherwise not available.

### Search strategies

We will develop comprehensive systematic search strategies to fulfill the demand of conducting systematic reviews according to predetermined protocols and established guidelines for meta-analyses of observational studies in epidemiology (the MOOSE group [14]).

This means striving to identify all relevant publications (high recall) and simultaneously, to identify only relevant publications (high precision) and thus yielding a low number needed to read (NNR) (minimizing subsequent workload). The information retrieval will be based on a search model structured by PICO components (see Additional file 1). Some snowballing and berrypicking strategies will be added to obtain a sufficient search yield. The search strategies will be adapted and processed by using the database-specific controlled vocabularies (MeSH, EMTREE) and additional free text terms. Included search terms will be e.g., amputation, amputee (search component “intervention”); lower extremity, foot, feet, limb, etc. (search component “problem”); and epidemiology, prevalence, incidence, frequency, population survey, survey data, administrative data, community data, etc. (search component “epidemiologic studies”). The search terms and components will be combined by using Boolean operators and, when possible, by proximity operators. The search protocols will be published in a transparent and reproducible manner.

All database records yielded by the search strategies will be exported into EndNote, where remaining duplicates will be removed manually.

### Study selection process and inclusion and exclusion criteria

Two authors will independently screen abstracts and titles of all references in order to identify original research reporting the incidence of LEA on the basis of inclusion criteria. We will exclude at this stage those studies that are clearly not population-based, or are randomized controlled trials, or which include only traumatic or tumor-related amputations, or report only the prevalence of amputations. Subsequently, two reviewers will independently screen the full-text articles of abstracts identified in the first phase.

We will include original full-text articles if they meet the inclusion criteria with respect to types of study, types of population, and the main outcome, regardless of the time period and year of publication of the study, definition of LEA, type of diabetes, age and sex distribution, and ethnicity. We will exclude studies if they report solely incidences of LEA among persons with DM referring to the total population (diabetic and non-diabetic) and not exclusively to the diabetic population. We also will exclude studies if they are published in a language other than English.

We will resolve disagreements pertaining to the inclusion of articles by consensus, involving of a third party if necessary.

### Data collection process

First, we will develop a data extraction sheet (based on the Cochrane Consumers and Communication Review Group’s data extraction template [15]). After that, we will carry out a pilot test using five randomly selected, included papers and then will refine the data extraction sheet accordingly. One review author will extract the following data from included articles, and the second author will check the extracted data. The two review authors will resolve disagreements by discussion; if no agreement can be reached, we plan to involve a third author who will then make a decision. We will use the most comprehensive data if several articles report data from the same study. We will contact the authors of included studies for explanation of anything that is unclear.

### Data items

We will extract the following information from each included article: (1) sources of data, (2) study design and study period, (3) populations (diabetic and non-diabetic population) and number at risk, (4) definitions of event (LEA) including reported incidence as well as severity criteria for amputations (major, minor, total), (5) type of diabetes (T1DM, T2DM, other types, all DM without distinguishing), and (6) absolute numbers and incidences of LEA.

### Data description

In studies with sufficient information on incident amputations, we will assess the outcomes of interest dependent on reported incidence measures (IR or CumI), relative risk of amputations comparing the diabetic to non-diabetic populations, cause of LEA, and type of diabetes. We will recalculate the reported IR per 100.000 person-years, if originally not reported as such.

We will categorize the following specific IRs: (1) amputations in individuals with diabetes among the population with diabetes, in comparison with (2) amputations in individuals without diabetes in the population without diabetes. Additionally, we will assign selected studies into three groups: (1) studies that count only the first or most severe observed amputation i.e., one amputation per person would be carried out, (2) studies reporting incidence of LEA on the basis of hospital data, and (3) studies counting all amputations.

### Statistical methods

From previous experience with a similar review [16–18], we expect studies to be too heterogeneous to allow for a quantitative summary of results. If, however, we find subsets of studies to be homogeneous enough, we will perform meta-analyses for incidence rates by Poisson generalized linear mixed models (GLMM) as recommended by Trikalinos et al. [19]. To account for estimation uncertainty, all statistical estimates will be given their 95 % confidence intervals. In any case, heterogeneity will be judged with regard to content and not by performing prior statistical tests for homogeneity.

### Data quality

Two independent reviewers will evaluate the quality of the included studies using the Cochrane approach study quality guide [20].

### Assessment of risk of bias in individual studies

The reviewer NM will rate the quality of the individual studies while reviewer KT will verify it.

#### Outcome and study level

For each included study, we will assess features that could potentially bias the estimates of LEA. Using this tool, we will rank potential sources of bias into low or high risk of bias according to the recommendations of the Cochrane approach (Table 1 [20]).

**Table 1:** Assessment of risk of bias (adapted to Cochrane approach Study Quality Guide [20])

Assessment items	Lower risk of bias	Higher risk of bias
Measurement and definition of outcomes LEA	Precise definition and description of how the LEA were recorded	No definition and description of how the LEA were recorded
Diagnostic criteria of diabetes	Documented by physician (clinical diagnosis, ICD)	Self-reported DM
Statistical methods: IR, CumI, RR	Presented as age-sex adjusted estimates; reported with CI	Crude rates; reported without CI
Time trends	Time trends reported using multivariate regression models	Time trends reported only descriptive
Duration of the observation period^a^	5 years and more	Less than 5 years

*CI* confidence interval, *CumI* cumulative incidence, *DM* diabetes mellitus, *ICD* International Statistical Classification of Diseases and Related Health Problems, *IR* incidence rate, *RR* relative risk

^a^Relevant for studies reporting time trend

## Discussion

We will perform the proposed systematic review to analyze the incidence of LEA in the diabetic population and to compare incidence rates in the diabetic and non-diabetic population.

One strength of this review is that the selection of studies will be based on a systematic search approach according to a predetermined protocol including clearly determined search strategies. Furthermore, we will use an extraction sheet based on the Cochrane Consumers and Communication Review Group’s data extraction template.

In conclusion, this review will help to summarize the available evidence for incidence of LEA. The publication of this protocol will contribute to making the search strategy, methods, and assessment of reviews transparent and accessible for all involved professional groups.

## Additional file

Additional file 1:
**Search model used to develop comprehensive systematic search strategies.**

## Abbreviations

CI: Confidence interval
CumI: Cumulative incidence
DM: Diabetes mellitus
GLMM: Generalized linear mixed models
IDDM: Insulin-dependent diabetes mellitus
ICD: International Statistical Classification of Diseases and Related Health Problems
IR: Incidence rate
LEA: Lower extremity amputation
NIDDM: Non-insulin-dependent diabetes mellitus
RR: Relative risk
