# Supplementary figures and images for: Incidence of lower extremity amputation in the diabetic compared to the non-diabetic population: a systematic review protocol
Source: Syst Rev. 2015 May 23;4:74. doi: 10.1186/s13643-015-0064-9 (PMC4450496; doi:10.1186/s13643-015-0064-9)

**Additional file 1: Search model to search databases**

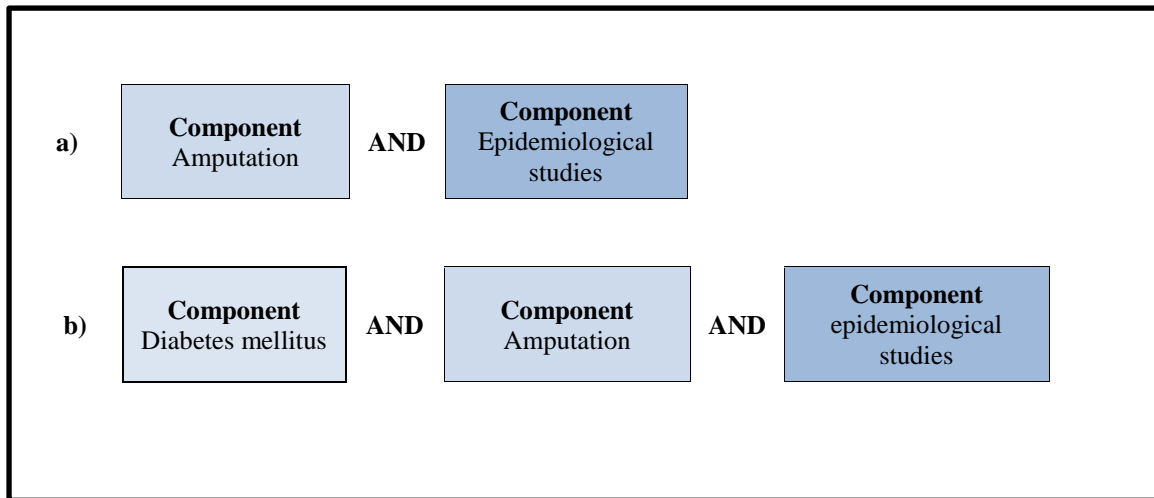

Supplement: Additional file 1: — Search model used to develop comprehensive systematic search strategies. [file 13643_2015_64_MOESM1_ESM.pdf]
